# A Mobile-Based Patient-Centric Passive System for Guiding Patients Through the Hospital Workflow: Design and Development

**DOI:** 10.2196/14779

**Published:** 2019-07-22

**Authors:** Chalee Vorakulpipat, Ekkachan Rattanalerdnusorn, Soontorn Sirapaisan, Visut Savangsuk, Natsuda Kasisopha

**Affiliations:** 1 National Electronics and Computer Technology Center Pathumthani Thailand

**Keywords:** user-centered design, health care informatics, mobile computing, data analytics, mhealth

## Abstract

**Background:**

A hospital is an unfamiliar place to patients because of its style, atmosphere, and procedures. These hospital characteristics cause patients to become confused about responding to protocols, which slows down the procedural flows. Some additional information technology infrastructure facilities and human resources may be needed to solve these problems. However, this solution needs high investment and cannot guarantee an accuracy of information sent to patients. To handle this limitation, EasyHos has been developed to help patients recognize their status (for example, “waiting for an appointment at 11am“) during their stay in a hospital using all existing infrastructure and hospital data and without changing existing hospital's process.

**Objective:**

The objective of this study was to provide a design of the EasyHos system and the case study in hospitals in Thailand. The design is usable and repeatable for small- and medium-sized hospitals where internet infrastructure is in place.

**Methods:**

The EasyHos system has been designed based on existing infrastructure, hospital data and hospital processes. The main components include mobile devices, existing hospital data, wireless communication network. The EasyHos was deployed at 2 hospitals in Thailand, one small and the other with a medium size. The experimental process was focused on solving the problem of unfamiliarity in the hospital. The criteria and pretest conditions regarding the unexpected problem have been defined before the experiment.

**Results:**

The results are presented in terms of criteria, pretest conditions, posttest conditions in the hospitals. The posttest conditions show the experimental results and impact of the system on users such as hospital nurses/staff and patients. For example, the questions from patients were reduced by 83.3% after using EasyHos system while nurses/hospital staff had 5 min more to do their routine work each day. In addition, another impact is that hospitals can create new information values from existing data, which now can be visible and valuable to patients.

**Conclusions:**

Hospitals' unexpected problems have been reduced by the EasyHos system. The EasyHos system has been developed with self-service and patient-centered concepts to assist patients with necessary information. The system makes interaction easier for nurses/hospital staff members and patients working or waiting in the hospital. The nurses/hospital staff members would have more time to do their routine works. Hospitals can easily set up the EasyHos system, which will have a low or nearly zero implementation cost.

## Introduction

Several hospitals encounter certain problems, such as the size, place, lack of available examination rooms, management system, and budgets. The differences between a hospital and patient’s home can cause confusion to patients. A hospital is an unfamiliar place for patients because of its style, atmosphere, and procedures. These hospital characteristics cause patients to become confused about responding to protocols, which slows down the procedural flows. Moreover, each hospital has different protocols for their procedures to maintain orderly patient flow in the hospital. For instance, different queue management systems are deployed in various hospitals. Typically, the queue is addressed by the nurse, and in some hospitals, it is displayed on monitors installed on the walls. The massive crowds waiting in the foyer of the examination room area indicate that these queue management systems do not work effectively. In particular, the level of sound coming from conversations in a crowded waiting room prevents the announced queue information from being heard. Furthermore, the queue display on the monitors is available only in specific areas. Therefore, patients are obliged to stay in the foyer until they are at the head of the line. Different solutions have been proposed, such as increasing the resources, evaluating demand management, performing operations research [1,2], redesigning the ideal procedure [3], and dividing the services into specialized units. Examples of these include chest pain observation units [4], rapid assessment zones [5], and clinical decision units [6]. Despite previous efforts, limited scientific knowledge has been obtained regarding approaches to improve patient flow [7].

To assist patients and reduce the hospital staff’s frustration, a system that encourages self-service concepts for the situation is a solution. Self-service is defined as the practice of serving oneself. Another term that relates to self-service is *customer self-service* (CSS). CSS defines types of electronic tools and items that support users who must obtain access to information and perform routine tasks using technology without requiring human assistance. Technologies that support the CSS model include software apps and kiosks. Software apps allow customers to interact through a software program on a mobile device to perform specific tasks such as internet banking. Developing a more self-service and patient-centered type of system that provides patients with their status and information could alleviate the problem of unfamiliarity and assist with overcrowding in a hospital [8,9]. Thus, the development of EasyHos, with its self-service and patient-centered concepts, would alleviate these problems for patients.

The EasyHos system was developed as a mobile phone app. This system was purposely developed with self-service and patient-centered concepts for patients staying at the hospital. It is simple and effective, with a user-friendly graphic user interface. The necessary patient information and guidance are provided and displayed on the EasyHos system, such as the examination room number, queue monitoring, the duration of stay, and the amount of the medical bill payment. The EasyHos app assists the patient in queue monitoring and notifies them when they move to the head of the line through a mobile phone. The EasyHos system allows patients to move around the hospital using the hospital’s map and directions. Patients can return on time to the location of their service based on the queue information provided, without the need to jump ahead in the line. The system assists patients immediately and at any time and frequency that the patient wants. The updates for the patient information are performed automatically in a passive approach by the EasyHos system. Thus, patients are satisfied by receiving the information that they request. Consequently, the hospital staff will be relieved from stress and will be able to perform their routine work more efficiently because the patients do not need to continually inquire about their status. The EasyHos system is necessary to address these issues.

This study aimed to present a tool, called the *EasyHos* system, as a strategic resource to reduce the unexpected problem at hospitals. This tool has been designed to improve the patient flow and prevent a delay in hospital services.

### Literature Review

The literature on health care technology exists in various forms. The health care and medical systems’ perspectives mostly address technologies such as location-based services (LBSs) [10], automated patient management systems [11], patient tracking tools [12], and complex event processing [13]. LBSs such as the global positioning system (GPS), Wi-Fi positioning systems, and Bluetooth Low Energy (BLE) and radiofrequency identification (RFID) technologies have been designed for indoor and outdoor navigation. The Connexient [14], Locatible [15], StandleyHealthcare [16], and SmartIndoor [17] technologies use the Wi-Fi fingerprint, BLE Beacon/iBeacon, or Handset Sensor Fusion to navigate, track inside a building, and provide indoor maps. All these tracking systems share the common purpose of determining a patient’s location in a hospital. These wireless location tracking technologies are key components for implementing an indoor tracking system.

The patent literature US2006/0011941A1 Automated Patient Management System [11] describes a patient activity management system. This technology allows patients to track their own status. The system allows users to view patient information on hospital servers through a kiosk or their own device using wired and wireless connections. This patent has objectives similar to those of the EasyHos system. Moreover, the output of this system is also similar to that of the EasyHos system because it presents patients with status information. Nevertheless, this technology relies on humans to enter the information into the system. Then, the system operates automatically. The limitation of this system is its reliance on human input because humans can cause errors and delays in entering the information. Another limitation of this system is the inflexibility of the process to support the hospital workflow. The hospital often improves its service by adjusting its workflow. To use a new workflow in the system, all the devices must be reset and the data must be re-entered. In contrast, the EasyHos system does not require data to be entered by humans and contains a flexible process. For this reason, the EasyHos system is more accurate, with 0% error. In addition, the EasyHos system supports the hospital’s workflow by using data analytics in processing the patients’ information according to the hospital conditions. The EasyHos system would be correlated with the hospital information system (HIS); therefore, if the hospital’s workflow changed, the system would automatically adjust without resetting each device.

A context-aware service is another technology that is used to track and provide an indoor map. The context-aware service requires more information from different sources. Bardram et al [18] deployed Bluetooth tags for tracking doctors, hospital staff, patients, and clinical management and status such as operating room scheduling based on context and location information. The information regarding clinical management and status is generated once the doctors, hospital staff, and patients have interacted with the Bluetooth tag reader located at a specific location only. The Cisco Context-Aware Healthcare Solution [19] was developed to locate patients and nonhuman assets (eg, devices and instruments) using radio frequency identification (RFID) tags interacting with readers. Another research study conducted on “A Context-Aware framework for patient Navigation and Engagement” [20] used a rule-based approach in a context-aware study. The reviewed literature aimed to provide suggestion information to patients. This study analyzed the context information, including the location, patient’s personal data, patient’s symptoms, and disease diagnosis, using numerous preset rules and conditions before providing the finalized information suggestion to the patient. This literature has greatly depended on location tracking technology such as the GPS.

The reviewed studies considered event-driven systems, which emphasized the deployment of an active-driven approach to acquire information based on event data or context data. The event-driven approach has been widely used, as presented in studies such as the following: *the Event-Driven* *Context Model In* *Elderly Health Monitoring* [21], *MyHealthAssistant* [22], *Healthcare-Event Driven Semantic Knowledge Extraction With Hybrid Data Repository* [23], and *Intelligent M2M: Complex Event Processing For Machine-To-Machine Communication* [24]. These studies used a specific mechanism, which was an active-driven approach based on an event-driven approach. An active-driven approach alludes to a user (tag device) intentionally moving closer to a sensor or reader. The active-driven feature can be a burden to users because they must make their way to the sensors or readers to obtain information. The limitation of these studies was the lack of budget and reluctance to make changes to the hospitals. Furthermore, the users were not incentivized to use the app tools in the actual environment.

The model in health monitoring of the elderly [21] adopted the event-driven approach for monitoring an elderly person’s movement using sensors to collect data. These movement data were analyzed for messaging notifications to the caregiver regarding whether the elderly patient needed an assistant or not. This research focused on the event of a single individual, which involved a small amount of data. In addition, the sensors and other hardware were used to help obtain data faster and were not as challenging as merely performing data analytics. Thus, to obtain accurate data, the sensors and experts would be required to set up and monitor the sensors closely.

MyHealthAssistant is an event-driven middleware for multiple medical apps on a mobile phone app-mediated body sensor network; a study by Seeger et al [22] proposed a middleware architecture for connecting the data and the various sensors on the mobile phone. The research did not report any prediction result using data analytics. The research contained shortcomings in merging the data from various resources to create the event data model because it was in an experimental phase. In comparison, using the EasyHos system, we successfully implemented the event data model and adopted it in an actual hospital environment.

*Healthcare-Event Driven Semantic Knowledge Extraction With A Hybrid Data Repository* [23] has proposed the use of multiple sources of data for an analytics model by emphasizing a data storage design. The research had the same aim as the EasyHos system. However, the research used the public data service app programming interfaces and analyzed the patients’ data. On the contrary, the EasyHos system uses real-time data and real-time context analysis. This real-time context analysis technique is more challenging than mapping the data of patients’ symptoms and behaviors because the data frequently change. Moreover, the EasyHos system has an architectural design that supports data movements for constant patient notifications. This system also includes an interface and data storage method, which are necessary for the data movements. This study did not discuss the design of the interface and data storage method layer. However, this paper was released in 2014, and the EasyHos system was already a patented system, with the technology disclosed in the same year.

Intelligent M2M, which uses complex event processing for machine-to-machine (M2M) communication [24], is an approach that uses concepts similar to those of the EasyHos system to analyze data in a software-based architecture. With the M2M connection or the Internet of Things technologies used in this research, the connection between machines would have a fixed format, that is, the M2M configuration is inflexible in comparison with human-to-machine communication such as that used in the EasyHos system. The EasyHos system allows users to move around or change the events all the time, such as queue changes. Furthermore, the research only discussed the system architecture and not the data analytics. The paper also did not report the system’s shortcoming of not being used in a real-world environment. On the contrary, the EasyHos system has a clearly defined scope of designing the system to eliminate the constraint of hardware dependence, especially its capability of using any model of mobile phone. The EasyHos system design focused on not engaging any other hardware tools. The study did not mention the hardware that would be involved, such as sensors, but the researchers claimed that their system had a software-based architecture, which needs hardware to operate. The EasyHos system is designed to not interfere with workflows that are performed by humans.

In a hospital, massive amounts of information such as that related to the patient’s treatment, the patient’s place in line, and the duration of the service are consistently produced. Originally, all of the hospital’s information is internally used exclusively among the hospital staff. This information is never visible to the patients, as it is meaningless raw data from the patient’s perspective. An electronic health care system for personal electronic health records [25] would empower patients and enhance their communication with doctors through the system. These health care systems generally need the involvement of patients, and the systems themselves contain sensitive information about patients’ symptoms and treatments [26]. However, these data are related to a patient’s symptoms, the doctor’s diagnosis, and the medical treatments. Furthermore, the data in the system are not being analyzed, such as in the EasyHos system. The analyzed and transformed information would gain value by becoming exclusively available to a specific patient. The information that describes an event or the actions performed by entities is carefully considered. This information includes, for instance, patients’ names that are added to the waiting list, which doctors the patients will see, the patients’ status, and the time of patients’ check-in and check-out at each department. This information can be used to track or measure an event for patients visiting the hospital [27,28]. To be precise, EasyHos uses a rule-based approach in a context-aware analysis to track patients. This method has created a location tracking technology independent of other technologies, such as GPS.

Overall, most studies in the existing literature are hardware dependent and implemented location-based systems or event-driven systems. These systems require funding for the hardware and incentives for the users to actively acquire information based on the event data or context data. To save costs and effort, it is necessary to conduct research on event-driven systems with a passive approach. Using this approach, the users are not required to input data, and no additional location tracking technologies are required. The idea of deploying this passive event–driven approach in a hospital with the context based on the patients’ requirements is explained and discussed in the EasyHos Development section.

## Methods

The proposed system was objectively designed to assist patients and the hospital. The EasyHos app simply works by scanning the barcode on the patient’s hospital card. The hospital number (HN) verifies their identity. The EasyHos system connects to the hospital’s server and database view to retrieve a fraction of the patient’s context. Consequently, the system uses the context analytics of the patient to categorize and display an up-to-date set of information on the patient’s mobile phone. Furthermore, the mobile phone can display the locations of the patient passively from the context analytics. The data analytics is performed in association with preset rules and conditions throughout the system operation to provide information suggestions to the patient. Once connected to the hospital’s server, the nonsensitive patient information is extracted and displayed on the mobile phone. Thus, a patient’s confidential information is not accessed.

A total of 3 main components are implemented as the EasyHos app for patients: (1) mobile devices (mobile phone and tablet), (2) existing hospital data, and (3) a wireless communication network (Wi-Fi or 3G). For the hospital’s perspective, the EasyHos system can be implemented without an additional hardware investment. Moreover, the hospital does not have to change their existing business processes or change their routine to learn how to use the EasyHos system. Specifically, the EasyHos system adopts a passive approach that operates in the background.

**Figure 1:**
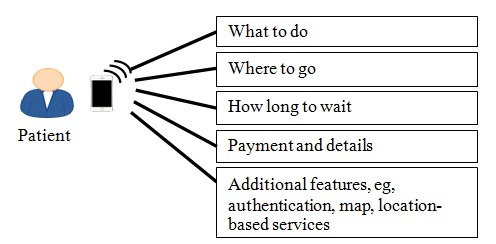
Example of information provided by EasyHos system to patient.

In addition to the passive approach, the system also uses data analytics techniques. The entire system provides context-aware service information based on event-driven data, such as where the patient must go, which doctor the patient will see, how long it will take to reach the head of the line, and how much the patient’s medical bill will be. The answers to these questions are displayed on the patient’s mobile device as part of the EasyHos system. Figure 1 shows a description of these activities. The answers to these questions are displayed on mobile devices with the information acquired from the HIS, including the patient’s personal data and service information. Personal data such as the patient’s full name, address, and HN number are involved. The service information presents the patient’s context that has been collected during their stay in the hospital. This service information includes the patient’s check-in time, the number of patients waiting for the same doctor, the patient’s service status, the medicine that the doctor prescribes, and the amount of the patient’s bill. These 2 types of data analyses use a rule-based approach with preset rules and conditions. As a result, a new, understandable, and suitable set of information items is generated for patients.

To obtain a clearer idea, the case of a patient named John is used to explain the data analysis mentioned above. For instance, John comes to a hospital and wishes to see Dr Bob. However, John did not have an appointment with Dr Bob at the hospital today. John arrives at the hospital at the information desk and asks what to do and where to go. The hospital receptionist asks John to scan a QR code of the EasyHos app system for installation on his mobile phone. At this stage, the EasyHos system checks John’s information in the HIS by scanning John’s HN card or identification (ID) card. Then, a message is generated and displayed on John’s phone, such as “John, please go to the medical record counter” to instruct John on what to do and where to go.

Once John has finished registration and made his appointment with Dr Bob, the EasyHos system checks for any changes that occurred to John’s record and show a message: “John, please wait for Dr. Bob at Room 2B.” The EasyHos system searches for the number of patients waiting for Dr Bob by searching all the records about *Dr Bob* and counting by filtering out who has a waiting status. For instance, there are 20 people waiting for Dr Bob. This acquired number of patients waiting in line is displayed on the mobile phone as follows: “John, there are 20 patients ahead of you at the moment. Please wait for Dr. Bob at Room 2B.” The number of people in line would decrease after a patient leaves the examination room. This decrease is because the doctor would update the HIS with the diagnosis, treatment, and prescription medicine information.

Normally, the HIS has features to calculate the total cost of the service and medical bill automatically. This calculation is for the convenience of the staff. As John is examined by Dr Bob, his information on the service and medical cost would be updated by Dr Bob. This information would be sent to the receptionist as well as to John’s mobile phone. On John’s mobile phone, a message such as “John, please go to the receptionist. Your total payment is 100 USD. Thank you.” is displayed. This message is the last message displayed to John for his hospital visit that day. The payment amount is displayed to avoid the surprise of overcharged fees. In addition, John can check with Dr Bob immediately if the bill is incorrect. This feature is to prevent wasting time for both the doctor and patient in checking the bill, correcting the bill’s details, and waiting at the receptionist again. Furthermore, John can prepare for the payment by withdrawing money from the automated teller machine because he knows the amount of the payment beforehand.

**Figure 2:**
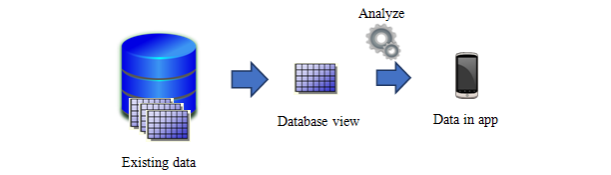
Process for extracting information from raw data.

The EasyHos system was designed to be personalized and context based for each patient. The new view table of existing data in the database management system was created. These view tables contain *only need-to-know* information for patients such as their full name, location, and place in line. Therefore, no patients can see other patient’s information. The EasyHos system does not access the HIS and database directly; it only accesses the necessary table’s extraction of the HIS. This access approach means that confidential information such as treatment methods and diagnoses are not violated. Then, a data query from the view table was developed to display the status of the patient on a mobile device, as shown in Figure 2. Therefore, the displayed message changes automatically when the context related to an individual changes or analyses are performed, as presented in Table 1 that shows the sample context between the HIS’s table view and the patient’s views.

In Table 1, the view of the HIS is presented on the left side. Data such as the patient’s name, doctor, register time, and status are extracted. These data are analyzed and become the information that is displayed on the patient view that is presented on the right side of Table 1. The context analysis was performed by looking at all the records and comparing them. For instance, to analyze how many patients are left before John can see Dr. Bob, 8 records are present in the view table, and the system searches for the patient named *John* and retrieves his record. Then, the system searches for patients who see Dr Bob and who have registered a time earlier or equal to John’s register time (10:16 am) to narrow the number of records searched. The search result leaves 4 patients on the list; they are *David, Jane, Albert, and John.* The next step is to see the statuses of all 4 patients who are still *waiting* and *in service*. In this case, there are 3 patients left. As David was examined by Dr Bob, he is no longer in the waiting queue. Jane, on the contrary, is in the examination room with an *in service* status; therefore, Jane would be the first person in line. Then, the next in line would be Albert, who has the status *waiting* and a register time before *10:16 am*. This information means that Albert is the second person in line. Thus, John would have to wait for 2 patients to see Dr Bob. This is the method used for data analysis.

**Table 1:** Data in a hospital view compared with the desired information in a patient view.

Hospital view (existing)				Patient view
Patient	Doctor	Register time	Status	What the patient should know
David	Dr Bob	10:00 am	Finished	David: go to cashier—how much?
Kim	Dr Roy	10:05 am	Finished	Kim: go to cashier—how much?
Jane	Dr Bob	10:07 am	In service	Jane: during treatment
Albert	Dr Bob	10:09 am	Waiting	Albert: see Doctor Bob—where? Wait 1 patient
Dan	Dr Roy	10:11 am	In service	Dan: during treatment
John	Dr Bob	10:16 am	Waiting	John: see Doctor Bob—where? Wait 2 patients
Peter	Dr Bob	10:30 am	Waiting	Peter: see Doctor Bob—where? Wait 3 patients
Ben	Dr Roy	10:31 am	Waiting	Ben: see Doctor Roy—where? Wait 1 patient

**Figure 3:**
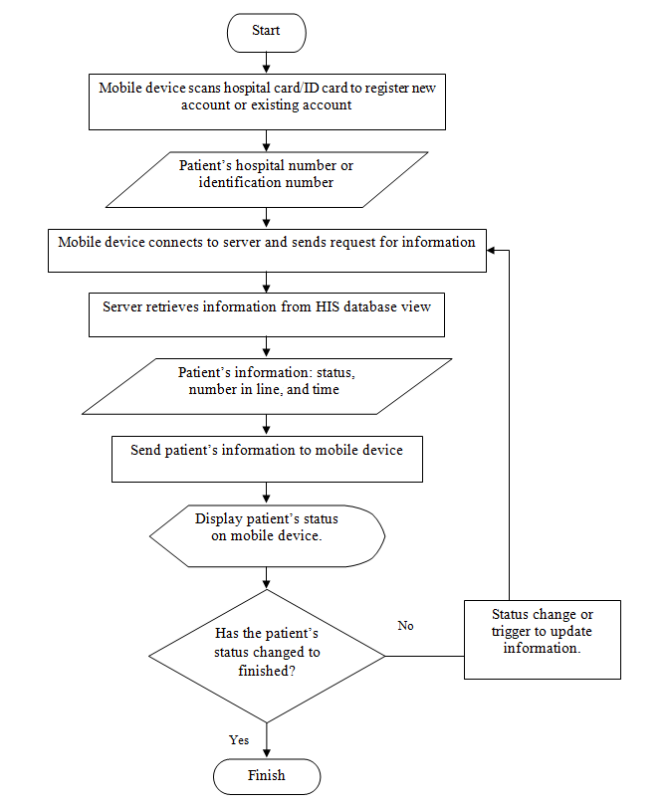
EasyHos system workflow on mobile application side. HIS: hospital information system; ID: identification.

The EasyHos software app development was performed using an Android operating system. The system was chosen because it is available for any mobile phone that has an Android operating system. Specifically, Android is a widely used operating system. The operating system is free and open source for the developer to create an app. The app designs the functionality that supports the scenario, which was explained at the beginning of the section. For a more comprehensive explanation, the workflow of the EasyHos System is drawn and displayed in Figure 3. The EasyHos mobile app starts working when a patient opens the app on their mobile phone. When the app fully starts, it asks the patient to scan a barcode on the hospital card for the HN. Alternatively, the EasyHos app can take the ID number on the ID card to retrieve the patient’s data instead. It should be noted that the HIS must record the ID number for patient ID verification when they are first registered. Then, the mobile apps are connected to the HIS and send a request with the HN or ID number.

Once the HIS has received the request, a new record regarding the new patient is created in the HIS’s database. Then, a new database view of the new patient is created according to the record in the database. At the request of an existing patient visiting the hospital, the patient’s information is retrieved from the database using the HN or ID number as the search condition. The results of the database search, with the patient’s information regarding their name, status, number in line, time of registration, and doctor’s name, are then selected and extracted for saving in the database view. This information is sent back to the mobile phone for displaying to the patient. Subsequently, the system constantly checks the patient’s status for any changes. The system is set to check for an update periodically, according to the configuration. However, if the patient’s status is shown as finished or equivalent to the completion of the hospital’s process, the EasyHos app terminates. Then, the system is ready for a new transaction for a new patient.

The EasyHos system provides flexibility for both patients and hospitals by adjusting the system to be suitable for both. From the hospital perspective, they can have different business processes or management systems. Even hospital wards can have different management systems. For instance, private and public hospitals have different business processes. This difference is because of the number of doctors and specialists available to patients. In a private hospital, there are more general doctors and specialists compared with a public hospital. Thus, the business processes and management systems are different, as the private hospital allows patients to directly see the specialist. On other hand, public hospitals lack specialists or even general doctors. For this reason, all the patients have to see the general doctor first although they want to see a specialist.

**Figure 4:**
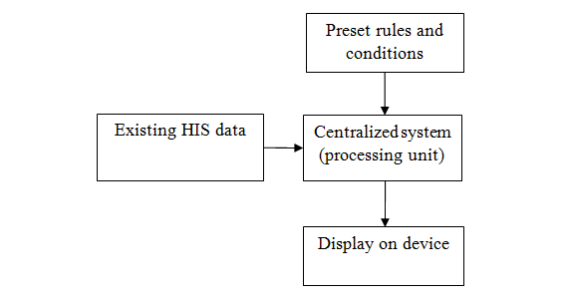
Overview of EasyHos system. HIS: hospital information system.

The mechanism behind the EasyHos system that improves services contains preset rules and conditions, a centralized system (processing unit) for data analytics, a graphical user interface on a mobile device, and the connectivity of a HIS. Figure 4 presents an overview of the EasyHos system, which shows the relationship of the components. Each component has different functionality and produces a different outcome. For instance, the centralized system analyzes the data from the HIS and the preset rules and conditions. The centralized system extracts only the necessary data on what the users must know from the HIS. Then, the new view table of the dataset is created and arranged in a structure such as with the XML or JavaScript object notation (JSON). Subsequently, the HIS is disconnected after the complete data extraction. The new dataset is used instead of being connected to the HIS all the time. This new dataset is used to prevent the confidentiality violation of the patient’s information and for the security of the information on a *need to know* basis.

**Figure 5:**
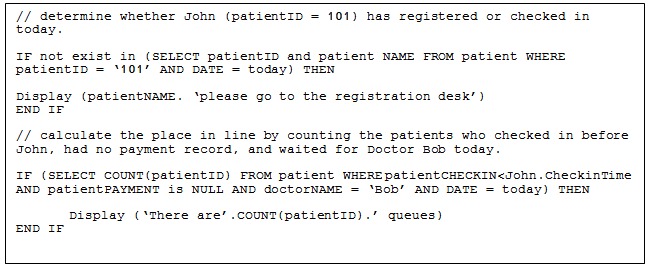
Example of pseudocode.

In the implementation process, the preset rules and conditions unit plays a vital role to drive the passive push of a patient’s information. The passive push of information is controlled by the centralized system. The push technology or server push is initiated by the centralized server, where the request for information to transmit to display devices occurs. Figure 5 shows an example of pseudocode that represents the preset rules and conditions. The preset rules and conditions are tied to the hospital business process. The consistency of the business process must be determined at the early stages to design preset rules and conditions. A hospital’s business process that is inconsistent or that continually changes may lead to difficulty in creating rules and conditions later.

**Figure 6:**
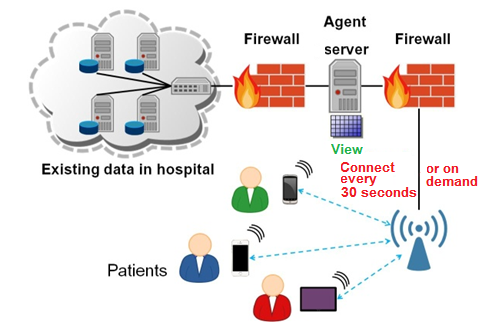
Process showing how patients use mobile devices to connect to hospital information system information.

Issues about privacy and security may be raised by patients. However, the proposed mechanism is designed by mirroring the existing database system and extracting only information about a patient’s activities, not sensitive data such as the date of treatment. People who do *shoulder surfing* cannot obtain patients’ identities and treatment information. In addition, the EasyHos system is designed to prevent hacking by having 2 firewall layers in its architecture. The process of patients using mobile devices to connect to the HIS information is shown in Figure 6. The setup of the firewall is placed in between the mobile device and the EasyHos system server. Another firewall layer is set up between the HIS and the EasyHos system server. Patients can retrieve their status on mobile devices by connecting to HIS information every 30 seconds or as a preset.

The EasyHos system uses rule-based data analysis to check the rule for each patient in the same context. The EasyHos system is designed on the basis of event data analysis. The system consists of the development of the preset rules and conditions, a centralized system (processing unit), a graphical user interface on a mobile device, and connectivity to the HIS. Thus, these elements are discussed in the Experiments and Results section. This section includes a discussion of a hospital’s pretest conditions and the posttest condition of the hospital after adopting the EasyHos system.

## Results

The EasyHos was objectively implemented, and an experiment was conducted for government hospitals. The EasyHos was deployed at 2 hospitals in Thailand, one small and the other with a medium size. EasyHos could also be deployed at a large hospital. However, this deployment was not conducted because of the unpredictable and complicated processes that affect the event data analysis using our EasyHos system. In addition, at large hospitals, patients often jump the line to see a doctor without registering their data in the computer system. Thus, a tool such as EasyHos would facilitate and solve this problem for both the patient and hospital.

The experiment with the EasyHos system focused on solving the problem of unfamiliarity in the hospital. During the operation trial, notes were accumulated statically. Moreover, the efficiency and technical aspects of the EasyHos app particularly focused on the delay in data retrieval, the accuracy of the data, and connectivity to the HIS server. The operation trial was performed for a 30-day period in a real hospital situation. The operation trial involved 10 nurses who carried mobile devices with an EasyHos system. However, kiosks were also available for some patients who did not have a mobile phone or had limited knowledge about mobile phones during the experiment. These actions were taken to assist the patients with their information, particularly for those who did not want to download and install the app or could not use the app for some other reason. Other components that were vital to setting up the EasyHos system were the HIS database and an internet connection. As mentioned earlier, the EasyHos system requires a connection to the HIS database using an internet connection through the hospital network.

Before the trial operation, the definitions of the criteria and pretest conditions regarding the unexpected problem were in place. The criteria were divided into 3 categories: (1) the statistics on queuing queries and related subjects, (2) information regarding what the patients wanted to know and related subjects, and (3) business process features. Table 2 lists the criteria and pretest conditions. In the first category, there were 3 questions related to the statistics for the queuing queries, as follows: (1) How often do patients ask nurses for their status per day? (2) How long does it take for the nurses to find answers and respond to patients? and (3) How often do the patients ask nurses about their place in line each day? These questions are listed in the first 3 rows of Table 2. In the second category, the questions were based on information that the patients wanted and related subjects, such as does the patient know what to do next or what information does the patient want while waiting and how many patients are not in the waiting area when the nurse announces their name. These questions are listed in the fourth to sixth rows in Table 2. In the third category, the question focused on the business process by looking at the error detection in providing patient services. The question is presented on the seventh row of the table.

**Table 2:** Criteria, pretest conditions, and posttest conditions in hospitals.

Criteria	Pretest conditions	Posttest conditions
How often do patients ask nurses for their status per day?	30 times	5 times
How long does it take for the nurses to find the answers and respond to patients?	10 min	5 min
How often do patients ask nurses about their place in line each day?	Average of 5 times per patient	1 time per patient
Does the patient know what to do next?	Patients do not know and keep asking constantly	Provide answers to patients immediately
What information do patients want while waiting to see a doctor?	How many patients are left?	None (EasyHos app provides patients with necessary information such as patient’s status)
	How long will it take until their turn?	—^a^
How many patients were not in the waiting area when the nurse called out their name?	5 people per day	On average, 1 person misses their turn per day. EasyHos notifies patients to walk back to the waiting area.
Error detection in patient services.	No validation process until confronted with the damage	EasyHos provides information to patients at all times. Patients are immediately notified of any changes that occur. This error can be seen by patients or the nurse at random.

^a^Not applicable.

The pretest conditions were the conditions that existed before the trial operation of the EasyHos system. The pretest conditions were used in collecting the data related to the defined criteria mentioned above. The pretest conditions are listed in Table 2. At a later stage of the experiment, the pretest and posttest were compared for the final result. The pretesting conditions were described for a situation with particular criteria. For instance, when a patient asked a nurse a question, it took approximately 10 min to obtain an answer. Each day, nurses had to answer questions an average of 5 times for a single patient. Furthermore, patients did not know what to do next or where to go. The most frequently asked questions that patients asked the nurses were *How many patients left until their turn?* and *How long until they could see the doctor*? Often, there were cases where a patient was not waiting in the waiting area when their name was announced. This was because of the long queues, which resulted in patients getting hungry or needing to go to the restroom. Finally, the hospital does not have an error detection procedure for patient services in the hospital business process. The nurses or hospital does not know about an error until after it occurs. There is no validation process available to detect errors in patient services because there is no record of each step when checking the status.

The posttest conditions present the hospital conditions after adopting the EasyHos system. The posttest conditions show the experimental results and impact of the system on users such as hospital nurses and staff and patients. In addition, another impact is that hospitals can create new information values from existing data, which now can be visible and valuable to patients. The posttest condition measures are related to the defined criteria, as presented in Table 2. For instance, patients would ask the nurses 5 times per day regarding their status. The EasyHos system helps nurses find the answer for patients by providing a patient’s information on a mobile phone, which uses 5 min of their time. With regard to the number of times patients have asked a nurse about their status, the number was reduced to one and then none per patient in each day. Usually, the patients do not know what to do next after each status and constantly ask the nurse. The EasyHos system provides the answer to the patient’s need immediately and constantly or as often as the patient needs. Providing this information also eliminates the questions that patients ask while waiting. The number of patients who are not waiting in the waiting areas was reduced to an average of 1 person missing their turn per day. This reduction was because EasyHos notified the patients to walk back to the waiting area. In terms of the error detection in patient services, the error could be seen by the patients themselves. The patients could monitor their status information at all times, and the system notified patients of any changes immediately.

**Figure 7:**
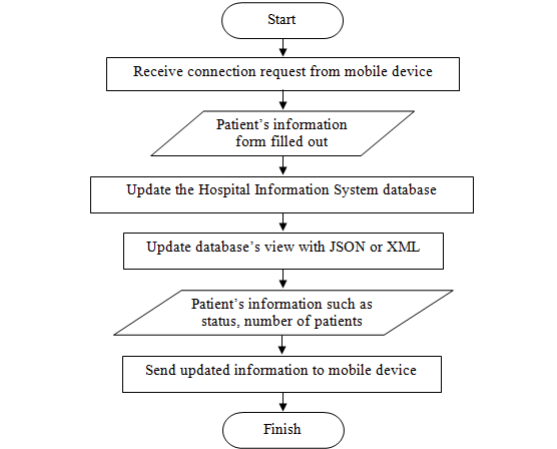
Example data flow of server side. JSON: JavaScript object notation.

During the experiment, the server was another main component needed for the EasyHos system to operate. The diagram portrays the procedures initiated with the hospital nurses and staff when filling out the patient’s information to retrieve their medical records. This process leads to updating the information on the hospital’s server, resulting in the standardized data in the database view (or JSON, XML) format being updated. The hospital server keeps updating the view continuously. The mobile device sends a connection request to the hospital server to transfer the patient’s information from the hospital database view. The hospital server remains connected to each other constantly to retrieve the updated data from the database view. The patient’s information such as their status and number of patients who are waiting for the same doctor is extracted and transferred to the mobile device. These are the procedures for requesting and transferring the patient’s information. The procedure that has been explained is described in the form of a workflow diagram and presented in Figure 7 as an example of the data flow on the server side.

**Figure 8:**
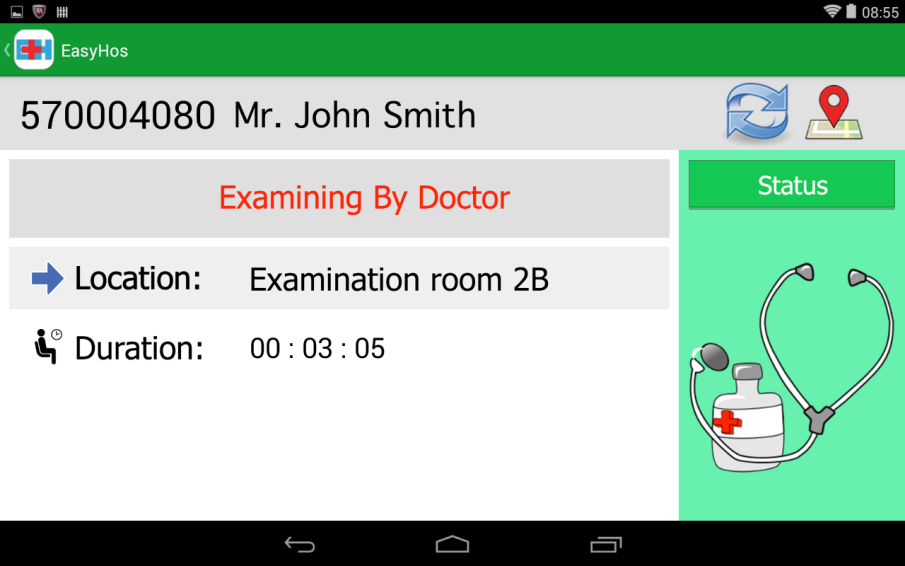
Example of EasyHos screenshot.

**Figure 9:**
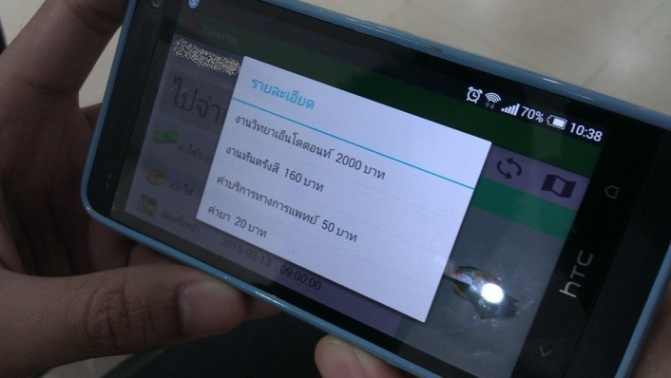
Real system showing billing details in Thai language.

In addition to the front end (mobile device) and back end (hospital server) that make EasyHos operable, the analyzed result would generate information on the mobile device by means of a user-friendly graphical user interface. Figures 8 and 9 show an example screenshot of the EasyHos system on a mobile phone device (display device). The screen consists of graphics, text, and an icon. The text on the mobile device normally appears in the Thai language. However, the picture display in Figure 8 depicts an example screenshot display on a mobile device that has been modified using the English language. The information includes the receptionist location, total fee, reimbursable fee, and total time spent in the hospital. Figure 9 depicts a real system displaying billing details, extended from the medical bill.

During the experimental period, the EasyHos system had some hindrances. These hindrances occurred as a result of a shortage of mobile devices because some patients did not have a mobile phone. In addition, some patients had limited knowledge about how to use a mobile phone and did not want to install the EasyHos app on their mobile phone. Furthermore, the developer confronted compatibility issues during the Android and iOS operating system integration on the mobile devices. Therefore, a new kiosk version is suggested to supplement the mobile device version. The kiosk version can use an EasyHos tablet version that is exactly the same as the mobile device version. The only change is the use of a front camera instead of a back camera to allow patients to easily scan a barcode. This change resolves the mentioned hindrance. The hindrance and evaluations are discussed further in the Evaluations section.

## Discussion

The experimental state of the EasyHos system is evaluated and discussed in this section. The results of the operational tests in 2 hospitals are shown with the correct information for patients because all the data involved in the analytics were based on existing data used on a daily basis. However, the system can send incorrect information to patients if the original data from the hospital system are wrong. In addition, it is clear that the hospitals did not require location tracking devices, as the proposed system uses only event-driven data extracted from the existing HIS. All these data were analyzed with preset rules and conditions, which finally created the information presented to patients based on the context or event. This system supports the passive approach in its communication to patients by pushing the information to users instead of users moving toward the readers or sensors. Table 3 presents a comparison of the existing approaches and the EasyHos system by emphasizing the criteria mentioned above.

The experiment involved a 1-month trial with real-field operation in a hospital. During the operation trial, 10 on-duty nurses performed the experiment. The equipment used for the operation trial included mobile phones and tablets for the nurses. This equipment was used to provide information to patients who did not want to install the app or did not have a mobile phone. During the experimental period, the criteria and pretest were defined before the operation test, as discussed in the Experiments and Results section.

The evaluation of the experimental results referred to the defined criteria. The number of pretest and posttest conditions present discussed here is the average number of criteria that occurred daily. The results of the evaluation of using the EasyHos system in responding to criteria are as follows: (1) the questions from patients were reduced by 83.3% after using EasyHos system, (2) nurses and hospital staff had 5 min more to do their routine work each day, (3) the patients rarely asked the nurses/hospital staff about their place in line and status, (4) the patients knew what to do and where to go from the information provided by the EasyHos system, (5) patients could go other places or do other things and relax while waiting on their doctor, (6) patients knew when to come back to the waiting area because EasyHos notified them and provided a map to assist in walking back to the waiting area, and (7) EasyHos had a double-check system for the hidden flaws in the process. The display information on a patient’s mobile devices would reveal any error that occurred in the process. Thus, the process had to be modified to resolve that error. Table 4 presents the criteria, pretest conditions, posttest conditions, and evaluation results.

As previously mentioned, the system has the limitation that the rules and conditions must be consistent, and all the data used for analyses are from the existing HIS. However, in many cases, especially in large hospitals, the rules and conditions can always change depending on the situation. Furthermore, if the data in the existing HIS are incorrect, the message generated from the system are also incorrect. Thus, the scenario mentioned is outside the scope in this study.

Security and privacy issues are also important concerns that should not be overlooked. People have expressed the concern that the proposed system may affect the HIS data. This concern involves the data extracted for data analysis from the HIS, which affects the system performance and availability of the HIS server. In addition, the HIS server is vulnerable to attacks if the EasyHos system allows more access to the HIS server. To reduce the vulnerability of the HIS, another server agent was deployed that only extracted and stored need-to-know data from the HIS server. Therefore, the HIS server is accessible only by this new server, and not directly accessed by end users.

In terms of privacy issues, sensitive data such as a national ID number or patient identity can be encrypted. The system may use 1-way encryption such as a hash function or other appropriate methods applied in a mobile environment [10]. In addition, encryption should be done end-to-end during transmission to ensure that sensitive data are not intercepted and understood by unauthorized persons.

After the experimental period, a full operation was conducted. The data collected over 6 months showed that the number of patients was 16,385 (average 2731 per month) at a small hospital and 25,238 (average 4206 per month) at a medium hospital. The uptime was 100%. The results on the data accuracy were not different from the trial because the accuracy depended on the existing hospital data.

However, during the trial and full operation, other hindrances were found. The cameras of mobile devices were unable or slow to read the barcode on the patient’s card. This issue was caused because the contrast setting of each camera is different when scanning the barcode. In addition, the barcode on the card is a traditional 1D barcode, which is an outdated technology. To resolve the problem, the traditional 1D barcode can be converted to a 2D code, such as a QFR. In addition, EasyHos has the flexibility to enter either the ID number on an ID card or a patient’s HN instead.

**Table 3:** Comparison with and results from other existing approaches.

Features and solutions	Location-based Services	Connexient	Locatible	StandlyHealthcare	SmartIndoor	Cisco Context-Aware Healthcare solution	The proposed scheme
Location or context based	Location based	Location based	Location based	Location based	Location based	Mainly location based and partially context based	Completely context based
Tracking hardware	GPS^a^	GPS	GPS	GPS	Bluetooth device	Tag and reader	Not required
Active or passive communication	Active	Active	Active	Active	Active	Active	Passive
Data analytic method	From location	From location	From location	From location	From location	From location	From rules and conditions
User interface/user experience design	Input from users required	Input from users required	Input from users required	Input from users required	Input from users required	Input from users required	No input from users required

^a^GPS: global positioning system.

**Table 4:** Evaluations regarding defined criteria.

Criteria	Pretest conditions	Posttest conditions	Evaluation results
How often do the patients ask nurses for their status per day?	30 times	5 times	The questions from patients have been reduced by 83.3%.
How long does it take for the nurses to find the answers and respond to patients?	10 min	5 min	Nurses and hospital staff have 5 min more to do their routine work each day.
How often do the patients ask nurses about their place in line each day?	Average of 5 times per patient. This is not including the patients checking on the medical folder placed in front of the examination room themselves.	1 time per patient	When using the EasyHos system, patients rarely ask the nurses and hospital staff about their place in line and status.
Do the patients know what to do next?	Patients do not know and keep asking constantly.	Provides answer to patients immediately	The patients know what to do and where to go from the information provided by the EasyHos system.
What information do the patients want while waiting to see a doctor?	How many patients left?; How long does it take until their turn?	None (EasyHos provides patients with necessary information such as patient’s status)	Patients can go or do other things and relax while waiting on their queue.
How many patients were not in the waiting area when the nurse called their name?	5 people per day	On average, 1 person misses their turn per day. EasyHos notifies patients to walk back to the waiting area.	Patients know when to come back to waiting area. EasyHos notifies patients and provides a map for patients to walk back to the waiting area.
Error detection in patient services.	No validation process until confronting the issue.	EasyHos provides information to patients at all times. Patients will immediately be notified of any changes that occur. This error can be seen by patients or any nurse.	EasyHos has a double check system for hidden flaws in the process. The display information on a patient’s mobile device would reveal the error that occurs in the process. Thus, the process must be modified to resolve that error.

In addition to the barcode issue, there was an incompatibility issue that occurred in one of the hospital systems. This error caused the information to be unsynchronized and absent from the HIS server. The hospital’s information and communications technology system had a workstation-based architecture, which meant other types of devices could not connect to the HIS. During the trial, appointments and notes were taken using a notebook to make it easier to move around; this was the reason that the data were not saved, and the information could not be displayed on a portable device. This constraint prevented unauthorized access to the HIS and tampering with the information. It was necessary for the nurse or staff to re-enter the recorded information on the hospital’s workstation again. This re-entering was to maintain the consistency of patient’s information in the HIS.

In terms of user feedback, most users, both hospital staff members and patients, were satisfied because of the ease of use and reduction of confusion. However, many users raised an issue about the shortage of mobile devices because some patients did not have a mobile phone. Some patients did not have a phone device that supported the barcode reader app. Some patients had limited knowledge on how to use a mobile phone, and some did not want to install the EasyHos app on their mobile phone. In addition, the developer confronted an issue with the mobile device operating system integration between Android, iOS, and Windows. As mentioned earlier, the EasyHos system was developed on the Android operating system. Therefore, patients were unable to install the app on an iOS operating system because of the different architecture and different programming language used. Therefore, a new kiosk version was suggested to supplement the mobile device version. The kiosk version can use an EasyHos tablet version that is exactly the same as the mobile device version. The only change is the use of a front camera instead of a back camera to allow patients to scan a barcode easily. This change resolves the mentioned hindrance. The evaluations are discussed in more detail in the Evaluations section.

### Recommendations for Future Work

This study has presented a new approach to reduce the unexpected problem in hospitals. The EasyHos system was developed with self-service and patient-centered concepts. The EasyHos assists patients by providing information about their place in line, notifying the patient when their name is almost at the top of the list, and providing the examination room number through a mobile phone. Patients can move around or do other things and come back on time to be seen by the doctor. The system also provides a hospital map and directions for patients in case a patient has lost their way. The system assists the patients immediately, at any time, and as often as the patient wants. The patients were satisfied to obtain the information they wanted passively and automatically. Consequently, the system has eased the workload of the nurses and hospital staff and makes it easier for patients waiting in the hospital. The nurses and hospital staff will have more time to do their routine work.

The EasyHos system has proved to be truly passive-oriented and secure and has a low additional cost. The EasyHos architecture was designed to support the scale of hospitals and assist hospitals in implementing the scheme without starting from zero. The system was designed to track the activities of patients without additional hardware devices such as readers or sensors. Therefore, no new hardware equipment is required, unless the hospital’s computer network infrastructure is insufficient. This method not only provides additional savings on hardware costs but also avoids business process modification. In addition, there are no new processes to be added to the hospital operation process. Furthermore, this advantage allows users to move freely without the need to use tagging with readers or sensors at a particular spot to obtain updated information. The system analyzes all of the activity data from the context or event extracted from the HIS to obtain information. The system does not directly access the HIS database but creates a database view that extracts necessary data. Thus, the information sent to patients would be private and accurate, because it is extracted from the HIS. Previously, hospitals were unable to disclose a patient’s information because of the confidentiality regulation. This policy prevents patients from seeing their own medical record. However, with the EasyHos system, hospitals have allowed the patient’s information to be disclosed at a certain level, which is valuable to the patient. However, this case depends on each hospital’s judgment about revealing information to the patients. By this action, hospitals have value added to the existing data.

During the experimental period, we found that the hospitals had inconsistent processes, with not many ad hoc processes or unexpected processes. For instance, some hospitals had policies and processes to allow special patients or priority patients, such as a military hospital giving priority to military personnel. In addition, other cases of priority and privilege in queue skipping occurred, such as with a monk, or patients who were over 70 years old and had first priority in the queue. The EasyHos system scope only supports ordinary processes, which do not include priority and privilege queues and VIP patients. In addition, the priority queue limits the EasyHos system operation in a hospital; therefore, the accuracy of the information sent to patients might be low. This is because the information sent to patients is fully reliant on the data from the HIS, which can be incorrect data. In other words, if the original source of information contains incorrect data, the extracted information that is sent to patients is also incorrect. Other hindrances that occurred during the experiment were the shortage of mobile devices, the shortage of knowledge on how to use mobile devices, and patients declining to install the EasyHos app. In addition, the developer confronted an incompatibility problem in the Android and iOS operating system integration on the mobile devices.

To resolve the aforementioned hindrances, a new kiosk version of the EasyHos system was suggested to supplement the mobile device version. The kiosk version uses the EasyHos tablet version, which is exactly the same as the mobile device version. The only change with the Kiosk version is the use of a front camera instead of a back camera to allow patients to scan a barcode easily. Moreover, the screen resolution may need to be adjusted to be suitable for the display screen.

**Figure 10:**
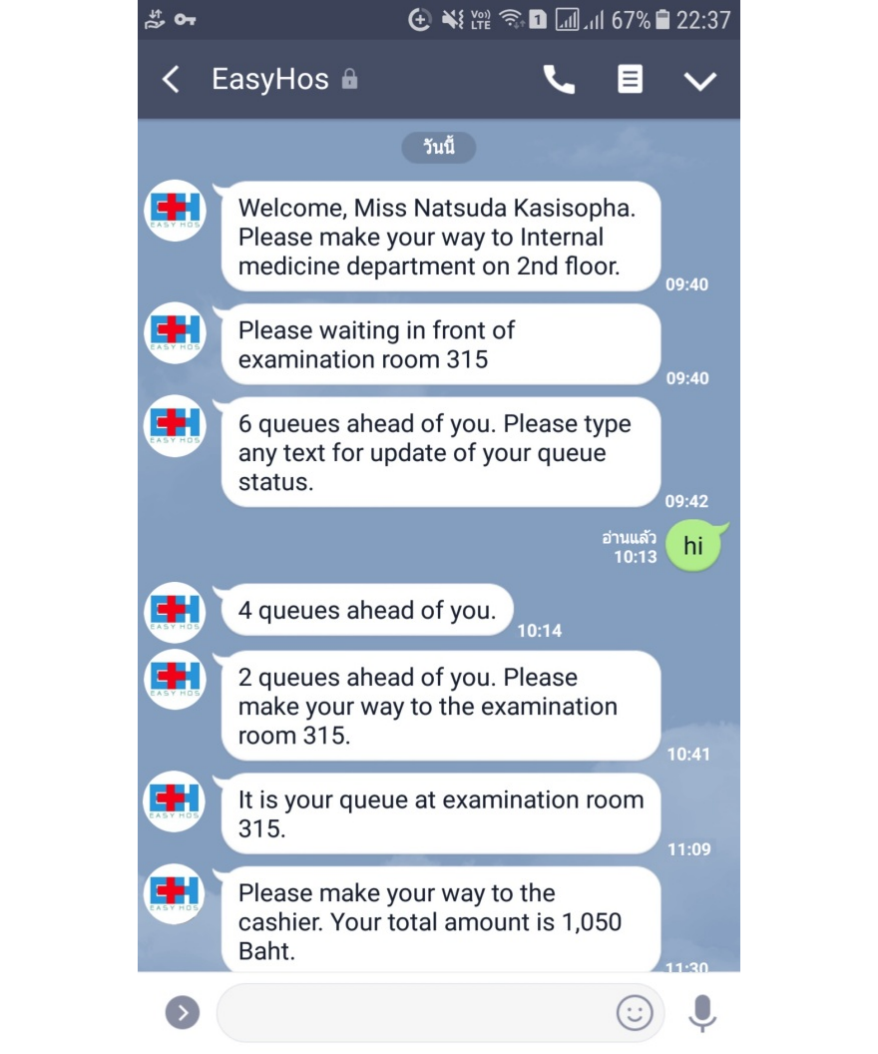
Example screenshot of Chatbot-like text from EasyHos system.

An additional solution to the hindrances would be the development of EasyHos as Chatbot, similar to existing messaging apps such as LINE [29] and WhatsApp [30]. The patients can add friends with EasyHos. Then, EasyHos continues sending updated information to the patients themselves. For instance, notification messages continuously pop up at the patient’s LINE account when there are updated. The notification message would be “Please wait in front of the examination room. There are two patients ahead of you in line” or “Please go to the receptionist. The total amount is 1,000 baht.” Figure 10 portrays the text message of Chatbot for the cross-platform of EasyHos that notifies patients on LINE messenger. The advantage of installing EasyHos on cross-platform messaging is that patients do not need to download and install the EasyHos app on their mobile device and can save more memory on mobile devices. Moreover, this method resolves the hindrance of the incompatibility between the Android and iOS operating systems on mobile devices.

In the future, we will create a database standard view that every hospital can use. The database standard view will allow a variety of different hospital database formats to work coherently with the EasyHos system. This feature will allow the EasyHos system to integrate with the hospital database no matter how complicated the hospital business processes are. The integration of the EasyHos system could be done easier and faster, and it will support various hospital database formats. The EasyHos development supports interoperability, allowing it to operate on top of various HISs. Thus, the architecture of EasyHos will be improved in response to the interoperability by extending it from a mobile app to a platform.

The EasyHos system in the sense of a platform will be able to operate as a centralized system (processing unit), where the system can work with different hospitals with different preset rules and conditions. In other words, EasyHos will be able to run on top of various operating systems and database formats. Therefore, new standards-based interfaces and open interfaces need to be defined to allow the app to interoperate on multiple hospitals’ platforms. This change will make EasyHos more effective by developing the EasyHos system to run as a platform as a service (PaaS) with a cloud architecture.

**Figure 11:**
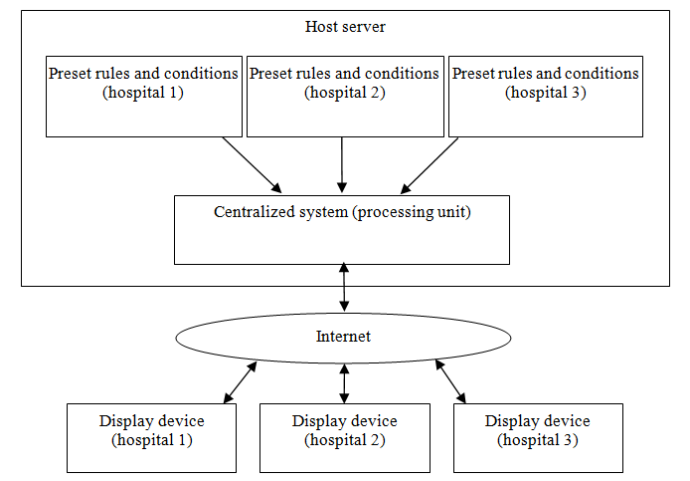
Platform as a service design of EasyHos.

The EasyHos system as a PaaS will allow the app to be executed and managed without the complexity of building and maintaining the infrastructure that is typically associated with developing and launching an app. Therefore, the reason for selecting the PaaS is to eliminate hindrances such as redesigning the software algorithm, server, and network infrastructure, which is time consuming. A hospital or service provider hosts software and hardware infrastructures, but, in the meantime, also provides a platform service that allows other hospitals to modify rules and conditions. In a real situation, other hospitals only use mobile devices (display devices) to connect to the host server to send a request or receive the suggestion information. In this case, hospitals may communicate with the host server using the internet through Wi-Fi, 3G and 4G, or a secured channel such as virtual private network. The architecture of the PaaS is shown in Figure 11.

Finally, the recommendations and future work for the EasyHos system will greatly impact hospitals by creating new information value from existing data. Furthermore, the recommendations will provide flexibility to hospitals, nurses and hospital staff, and patients and increase the efficiency for those entities.

### Conclusions

A hospital’s unexpected problem has been reduced by the EasyHos system. The EasyHos system has been developed with self-service and patient-centered concepts to assist patients with necessary information. The system makes interaction easier for nurses and hospital staff members and patients working or waiting in the hospital. The nurses and hospital staff members would have more time to do their routine works. The hospital can easily set up the EasyHos system, which has a low or nearly zero implementation cost. Because EasyHos fully relies on the data in the HIS, the limitation is that the data accuracy depends on the existing data stored in the system. Another version of EasyHos such as a kiosk or chatbot version would allow patients to use the system without installing an app on their own mobile device. Furthermore, a platform version of EasyHos would allow the system to operate as a centralized system (processing unit), where the system could work with different hospitals with different preset rules and conditions. This provides the alternatives and flexibility for patients and hospitals to use the EasyHos system.

## Abbreviations

BLE: Bluetooth Low Energy
CSS: customer self-service
GPS: global positioning system
HIS: hospital information system
HN: hospital number
ID: identification
JSON: JavaScript object notation
LBSs: location-based services
M2M: machine-to-machine
PaaS: platform as a service
RFID: radiofrequency identification
